# Projections of surface air temperature required to sustain permafrost and importance of adaptation to climate change in the Daisetsu Mountains, Japan

**DOI:** 10.1038/s41598-021-94222-4

**Published:** 2021-07-30

**Authors:** Tokuta Yokohata, Go Iwahana, Toshio Sone, Kazuyuki Saito, Noriko N. Ishizaki, Takahiro Kubo, Hiroyuki Oguma, Masao Uchida

**Affiliations:** 1 Earth System Division, National Institute for Environmental Studies, Tsukuba, Japan; 2 International Arctic Research Center, University of Alaska Fairbanks, Alaska, USA; 3 Institute of Low Temperature Science, Hokkaido University, Sapporo, Japan; 4 Research Institute for Global Change, Japan Agency for Marine-Earth Science and Technology, Yokohama, Japan; 5 Center for Climate Change Adaptation, National Institute for Environmental Studies, Tsukuba, Japan; 6 Biodiversity Division, National Institute for Environmental Studies, Tsukuba, Japan; 7 Department of Zoology, University of Oxford, Oxford, UK; 8 Durrell Institute of Conservation and Ecology (DICE), School of Anthropology and Conservation, Canterbury, UK

**Keywords:** Climate sciences, Atmospheric science, Climate change, Cryospheric science

## Abstract

Permafrost is known to occur in high mountainous areas such as the Daisetsu Mountains in Japan, which are located at the southernmost limit of the permafrost distribution in the world. In this study, areas with climatic conditions suitable for sustaining permafrost in the Daisetsu Mountains are projected using bias-corrected and downscaled climate model outputs and statistical relationships between surface air temperatures and permafrost areas. Using freezing and thawing indices, the size of the area in the Daisetsu Mountains where climatic conditions were suitable for permafrost were estimated to be approximately 150 km^2^ in 2010. Under the RCP8.5 scenario, this area is projected to decrease to about 30 km^2^ by 2050 and it is projected to disappear by around 2070. Under the RCP2.6 scenario, the area is projected to decrease to approximately 20 km^2^ by 2100. The degradation of mountain permafrost could potentially affect the stability of trekking trails due to slope displacement, and it may also have deleterious effects on current alpine ecosystems. It is therefore important to accurately monitor changes in the mountain ecosystem environment and to implement measures to adapt to an environment that is projected to change significantly in the future.

## Introduction

Areas with ground temperatures that remain below 0 °C for more than two years are referred to as permafrost^1^, and approximately one quarter of the Northern Hemisphere and 17% of the Earth’s exposed land surface is permafrost^2^. Permafrost is found in mountainous areas as well as in high-latitude tundra and taiga regions. The permafrost zone in mountainous areas currently constitutes 27–29% of all permafrost areas^3^. Recent observations have shown that the permafrost in mountainous areas is thawing in the European Alps^4–8^, Scandinavia^9,10^ and on the Tibetan Plateau^11^.

The degradation of permafrost in mountainous areas has been reported to increase the frequency of rockfalls^12,13^ and it may increase the frequency and scale of landslides^14–16^. Further, an increase in disasters in mountainous areas due to permafrost degradation threatens the safety of hikers and mountaineers^17,18^, and requires changes to iconic mountaineering routes and climbing seasons^19^. The thawing of permafrost can also have a significant impact on the alpine ecosystem through changes in temperature, soil moisture and groundwater^20–22^. The disappearance of permafrost, coupled with insufficient precipitation in summer and reduced snowmelt, may result in a lack of water during the growing season, changes in species compositions, and reduced greening and productivity^23,24^.

The islands that make up Japan form a long arc that extends from tropical regions in the south to Palearctic regions in the north. Permafrost has been reported to exist in the Daisetsu Mountains^25–28^, Mt. Fuji^29,30^ and Mt. Tateyama^31,32^. The Daisetsu Mountains on the northern island of Hokkaido, which are known to support a variety of alpine plants and animals, are also a valuable natural resource that is visited by numerous climbers every year^33–35^. However, there is some concern that the thawing of permafrost will have a major impact on this mountain ecosystem.

Although the thawing of permafrost can have a variety of impacts on human society and natural ecosystems^36–42^, it is a sub-surface phenomenon that cannot be easily observed remotely. As a result, the distribution and dynamics of permafrost are less well understood than those of glaciers or snow^3^. In particular, because areas of mountain permafrost are often difficult to access, their distribution in many mountainous regions can only be inferred^2,3^. Consequently, future projections of mountain permafrost distribution are limited and have been limited to the European Alps^7,8^ and the Tibetan Plateau^11^. Since air temperature is the major driver of permafrost dynamics, it is projected that the extent of mountain permafrost will decrease significantly during the twenty-first century due to global warming^7,8,11,38^. However, no such projections of future permafrost distribution have been undertaken in East Asia, including in Japan, to date.

As methods for projecting the future changes in permafrost, numerical global models (e.g., land surface, climate, earth system models), and fine-scale models (e.g., vertical one-dimensional models) can be used^3^. However, the resolution of these global models is about 100 km, which means that they are typically not well suited to describe, in detail, the complicated phenomena near the ground surface that are typically characterized by large inhomogeneities, or phenomena that have a spatial scale that is smaller than the resolution of the model^43–46^. In order to better understand the behavior of permafrost in mountainous areas, it is necessary to consider detailed spatial information, such as altitude^26,28,43–48^. On the other hand, fine-scale models, such as vertical one-dimensional models, can be used to estimate the ground temperature distribution at a defined point in detail^9,10^. However, the areal extent that the fine-scale models can deal with effectively is limited^3^, which means that estimating the extent of permafrost in a large mountainous area, such as the Daisetsu Mountains, is difficult.

As an alternative to numerical models for estimating the permafrost distribution, methods using statistical relationships based on proxy meteorological data, such as surface temperature, have been developed^49–52^. For example, Sone^27^ used freezing and thawing indices to perform a detailed analysis of the distribution of permafrost in the Daisetsu Mountains. Saito et al.^52^ performed detailed estimates of the permafrost distribution in East Asia by analyzing the relationships between the observed permafrost distribution reported by Brown et al.^53^ and freezing and thawing indices.

The actual distribution of permafrost is determined by complicated processes, such as the direction and angle of inclination, wind direction and speed, and snow cover and snow accumulation^28^. It is important to note that the results of methods for estimating the distribution of permafrost that are based on statistical relationships are only approximate^52^. However, using the available proxy data, statistical methods can be used to widely estimate the climatic conditions under which permafrost can exist, both spatially and temporally.

In this study, by applying the statistical method developed by Satio et al.^52^ for estimating the permafrost distribution to outputs from bias-corrected and downscaled climate model with a 1 km resolution^54^ for the area of interest, we investigated the climatic conditions required to sustain permafrost in the Daisetsu Mountains. In Japan, the Climate Change Adaptation Act was enacted in 2018, and Ishizaki et al.^54^ created bias-corrected climate scenarios with a 1 km resolution in order to support the formulation of climate change adaptation measures by various actors in Japan. In general, it is difficult to create reliable high-resolution climate scenarios because the numerical models contain errors, and meteorological observations can only be made at a limited number of points. Ishizaki et al.^54^ overcame these difficulties by correcting the systematic errors in climate model simulations based on detailed spatial meteorological observation data for Japan.

The goal of this study was to contribute to the formulation of strategies to address the various problems caused by permafrost thawing. To date, no future projections of the distribution of mountain permafrost based on the latest climate scenarios (Representative Concentration Pathways, RCP)^55^ have been published for Japan. In "Results and discussion" section, we first evaluate the effectiveness of the method by comparing the results obtained for current climate conditions with actual permafrost observations. Since observations of meteorology and permafrost have been conducted in the Daisetsu Mountains in Hokkaido since the 2000s, we used these observations to validate our estimates. In "Results and discussion" section, the future projection of climatic conditions suitable for the maintenance of permafrost is performed under a scenario that is close to the Paris target (RCP2.6); that is, that future global average surface temperature changes are maintained within approximately 2 °C compared to pre-industrial surface temperatures, and a scenario in which GHG emissions continue at the current pace (RCP8.5). In "Discussion and conclusions" section, the discussion and conclusions of the results are presented. Finally, "Methods" section describes the details of bias-corrected climate scenarios used for the analysis, and the methods used to estimate the climatic conditions that favor the maintenance of permafrost.

## Results and discussion

### Present annual mean surface air temperatures in the Daisetsu Mountains, Hokkaido

The Daisetsu Mountains comprise a group of volcanoes located at the center of Hokkaido, the second largest island of Japan. The mountain range consists of approximately 10 mountains, some of which are taller than 2000 m. Permafrost has been studied in the Daisetsu Mountains since the 1970s^56^, and continuous meteorological observations have been carried out since 2005^57^.

**Figure 1:**
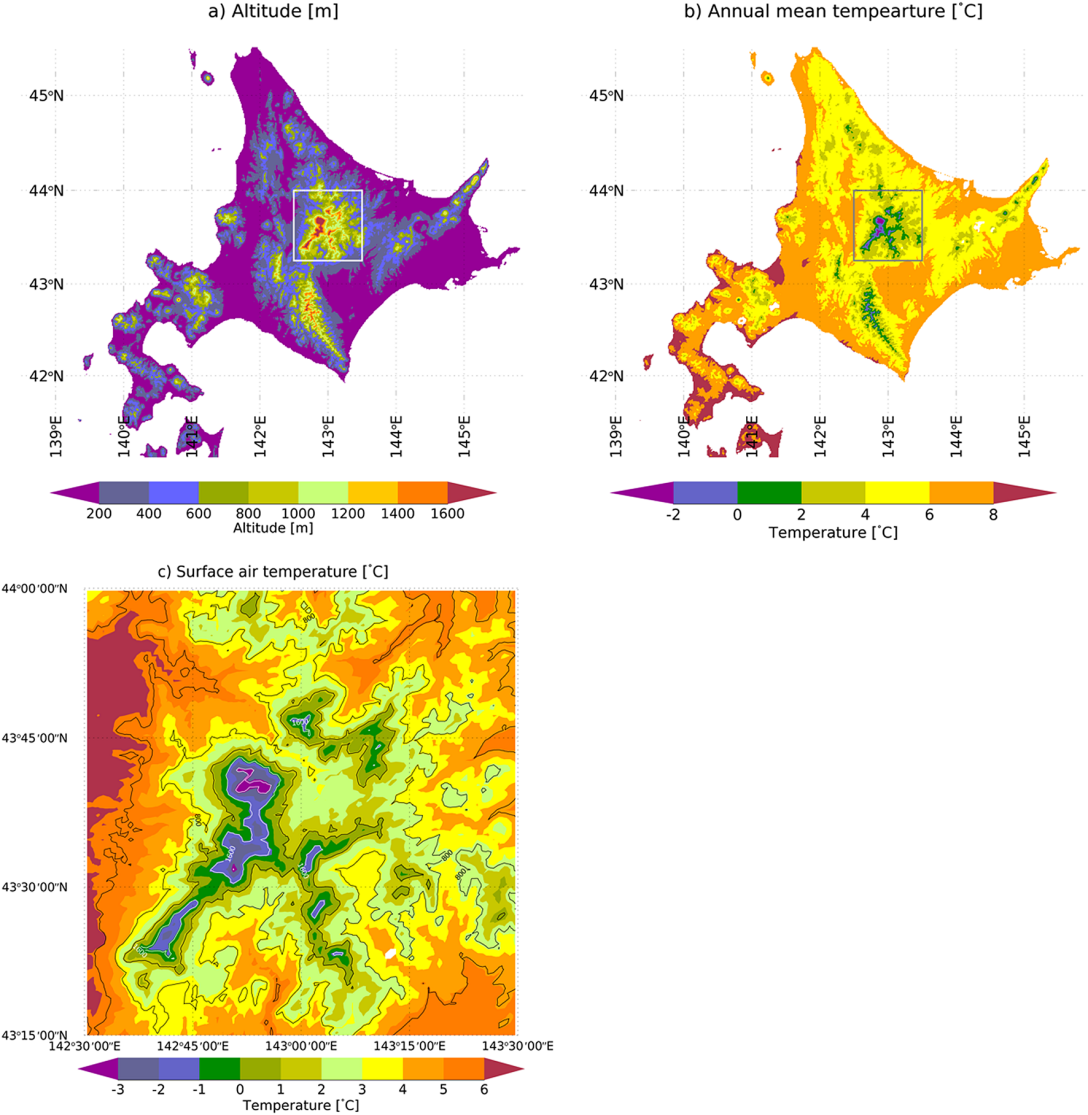
(**a**) Altitude distribution [m] and (**b**) annual mean surface air temperature [°C] under the current climatic conditions (2001–2010) in Hokkaido. The annual mean surface air temperature represents the average of the four bias-corrected scenarios of different global climate models. The inset shows the Daisetsu Mountains. The annual mean surface air temperature of the Daisetsu Mountains is shown in (**c**). IDL (https://www.l3harrisgeospatial.com/Software-Technology/IDL) is used to plot this figure.

Figure 1a shows the distribution of altitudes in Hokkaido. The data in the figure is based on Ohno et al.^58^, who generated a 1-km grid of observational meteorological data for Japan. The inset shown in Fig. 1a contains the Daisetsu Mountains, which are of particular interest in this study. Figure 1b shows the annual mean surface air temperatures under current climate conditions (average of 2001–2010) that were estimated using the bias-corrected and downscaled climate model outputs by Ishizaki et al.^54^. In that study, bias-corrected climate scenarios based on four global climate models (GCMs, see  "Methods" section) were generated; Fig. 1b shows the average annual mean surface air temperatures of the four GCMs. As shown in Fig. 1a,b, the Daisetsu mountains are the site of the highest altitude and lowest surface temperature in Hokkaido. Figure 1a,b also show that the annual mean surface air temperature is low in the central and mountainous parts of Hokkaido and higher near the coast.

Figure 1c shows the annual mean surface air temperature in the Daisetsu Mountains under current climate conditions (average of the four GCMs for 2001–2010). The annual mean surface air temperature in the Daisetsu Mountains decreases with an increase in altitude. At altitudes ≥ 1600 m, the annual average temperature is ≤ −1 °C. Conversely, at altitudes ≤ 800 m, the annual average temperature is ≥ 4 °C. As shown in Supplementary Figure S1, we confirmed that the annual mean surface air temperature (Fig. 1c) is consistent with that of the 1-km grid meteorological dataset for Japan^58^.

### Distribution of permafrost areas in the Daisetsu Mountains under current climate conditions

The freezing (thawing) index, defined as the cumulative daily temperature below (above) the freezing point of 0 °C, has been used as a proxy for inferring permafrost distribution^49–52^. Saito et al.^52^ used this index to develop a high-resolution (2 km) method to estimate the permafrost distribution in northeastern Asia, including the Japanese mountainous permafrost region investigated in the present study. We therefore employed the same method to infer the permafrost distribution using the bias-corrected, 1-km resolution climate scenarios developed by Ishizaki et al.^54^.

Saito et al.^52^ classified the permafrost regions in northeastern Asia into two categories based on the freezing and thawing indices: (a) Climate-driven permafrost (CDP), which are regions where climatic conditions favor the development and/or maintenance of continuous permafrost; and (b) Environmentally conditioned permafrost (ECP), which are regions where the presence of permafrost is conditional upon environmental factors, such as ecosystem characteristics, topography or geology^59,60^. In addition, Saito et al.^52^ also divided seasonally frozen ground into two subcategories: ground that undergoes (c) seasonal freezing (SF), and ground that undergoes (d) intermittent freezing (IF). These distinctions were made in order to distinguish between seasonal frost that is deep and/or persistent, and frost that exists for a short time, i.e., less than two weeks. The criteria used to classify the permafrost into the above categories are based on the freezing and thawing indices and are explained in "Statistical method for inferring permafrost distribution" section.

**Figure 2:**
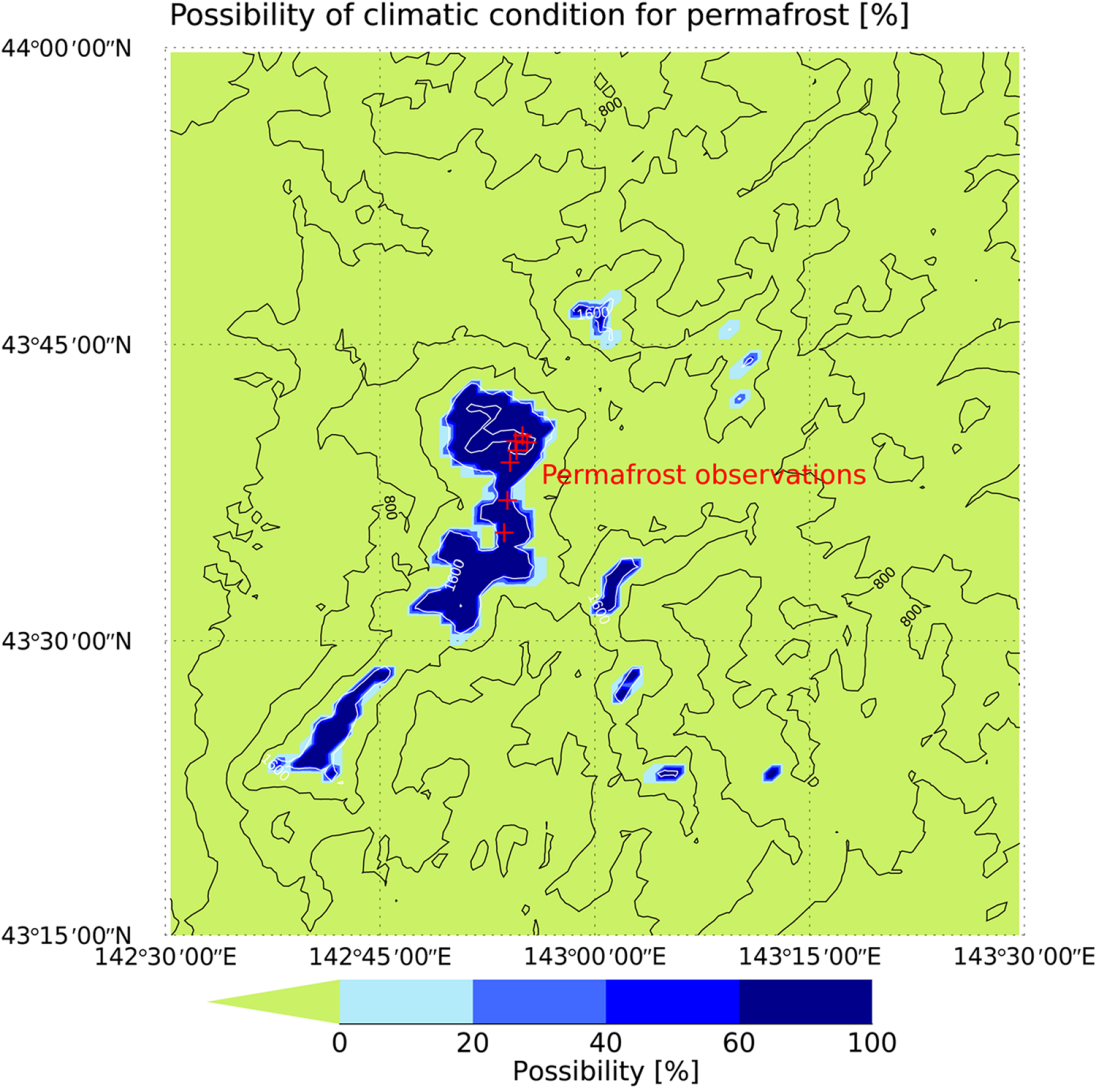
Location of climatic conditions favoring the development and maintenance of permafrost in the Daisetsu Mountains in 2010 under current climate conditions [%]. Contour lines show altitude distribution. The environmentally conditioned permafrost (ECP) region is derived based on the probability determined based on the four bias-corrected climate scenarios. Red points indicate the places where permafrost has been observed (see Supplementary Table S1). IDL (https://www.l3harrisgeospatial.com/Software-Technology/IDL) is used to plot this figure.

Figure 2 shows an area with climatic conditions that are suitable for permafrost development in the Daisetsu Mountains. The areas shown are probable locations of permafrost determined using the four bias-corrected climate scenarios in each grid. For example, if a grid is designated as containing permafrost in two of the four climate scenarios, then probability of permafrost in that grid will be 0.5. Saito et al.^52^ classified permafrost into two types (i.e., CDP and ECP); however, based on the freezing and thawing indices, only ECP was present in the Daisetsu Mountains. Given that the actual distribution of permafrost is determined by complex interactions between environmental phenomena, such as topography and geology^28^, the grid cells classified as permafrost in this study only indicate where the climatic conditions are suitable for the maintenance of permafrost. We refer to areas containing these grid cells as “ECP regions” hereafter. In addition to surface air temperature, snow cover is an important factor affecting the distribution of permafrost. In general, permafrost is distributed in wind-blown gravel areas where the snow cover is typically thin. Due to the limited distribution of wind-blown gravel areas, the actual distribution of permafrost would be smaller than the area estimated using only surface air temperatures^27,28^.

As shown in Fig. 2, the probability of an ECP region is high in areas above 1600 m. Depending on the bias-corrected climate scenarios, even areas below 1600 m are identified as potential ECP regions. Figure 2 also shows areas where permafrost was confirmed by previous observational studies and Supplementary Table S1 lists the coordinates and references of the sites where permafrost was confirmed. Permafrost has been observed at altitudes above 1600 m^28,57,61–63^, which is consistent with the findings of the present study (Fig. 2). We confirmed that the annual mean surface air temperature at these field observation sites is consistent with the observational data, as shown in Supplementary Figure S2. Even though the average of bias-corrected climate scenarios slightly overestimate the annual mean surface air temperature, the observational data falls within the range of the climate scenarios.

As can be inferred from Fig. 1, the high altitudes and low air temperatures in the Daisetsu Mountains make it very difficult to conduct physical observations. As a result, the number of locations where permafrost has been confirmed is very limited. The reliability of the method presented in the present study could therefore be improved if the number of observation points are increased in the future. Figure 2 will also be useful when considering candidate sites for such observations.

### Future projections of climatic conditions suitable for the maintenance of permafrost in the Daisetsu Mountains

**Figure 3:**
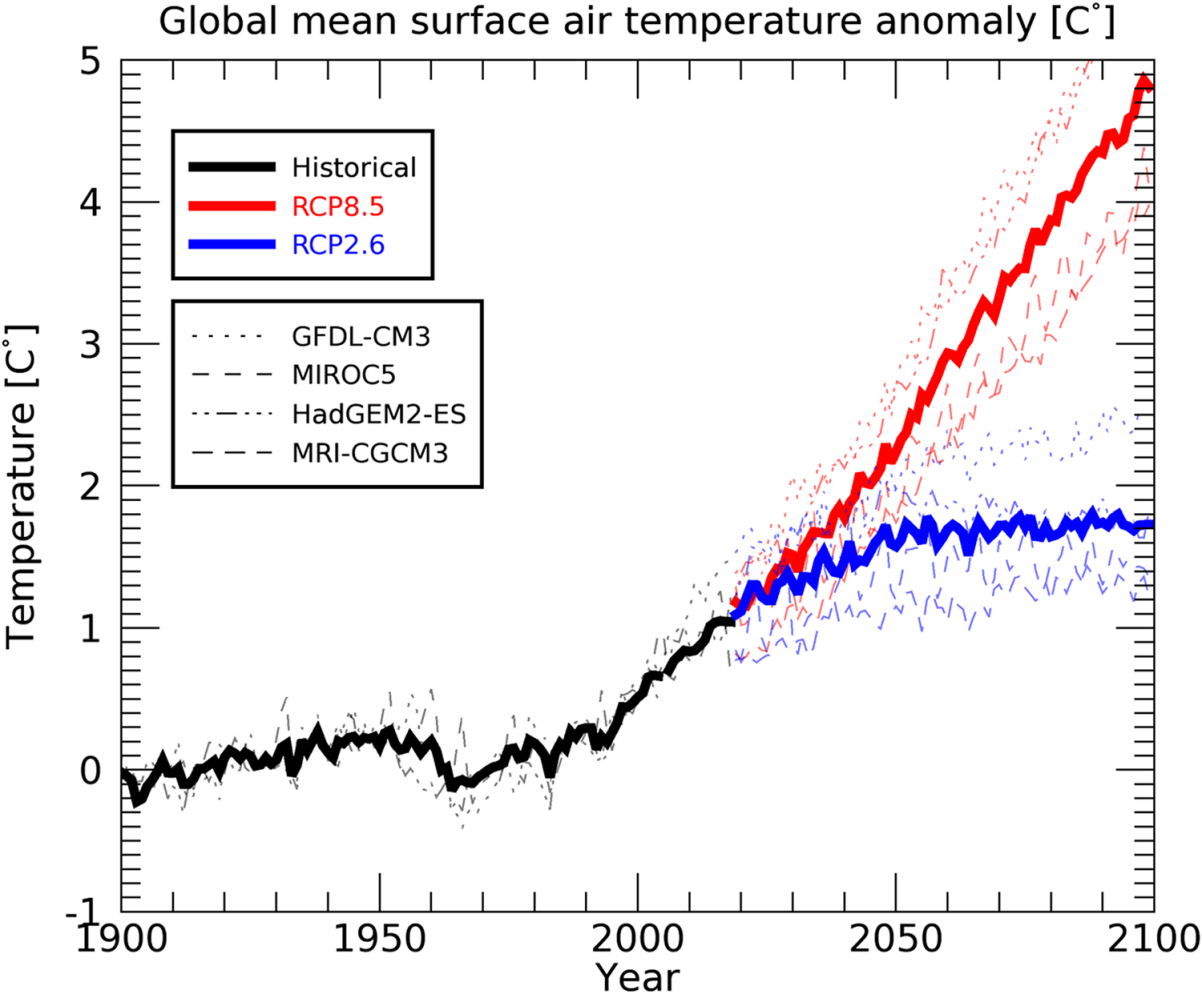
Time series of global mean surface air temperature for the historical (black), the RCP2.6 (blue), and RCP8.5 (red) scenarios. Thin broken lines show the results of the four global climate models (GCMs), and the thick line is the average of the four GCMs. Anomalies from the baseline (defined as the average of the 1900–1930) are shown.

This section discusses the results of future projections of permafrost coverage. Figure 3 shows the changes in the global mean surface air temperature estimated by the four bias-corrected climate scenarios. Under the RCP8.5 scenario, temperatures are projected to continue to rise throughout the twenty-first century. Compared to current levels, global mean surface air temperatures are projected to rise by about 4 °C by the end of the twenty-first century. On the other hand, under the RCP2.6 scenario, the global mean surface air temperatures are projected to stabilize by around 2050 and global average temperatures are projected to remain constant thereafter (Fig. 3).

**Figure 4:**
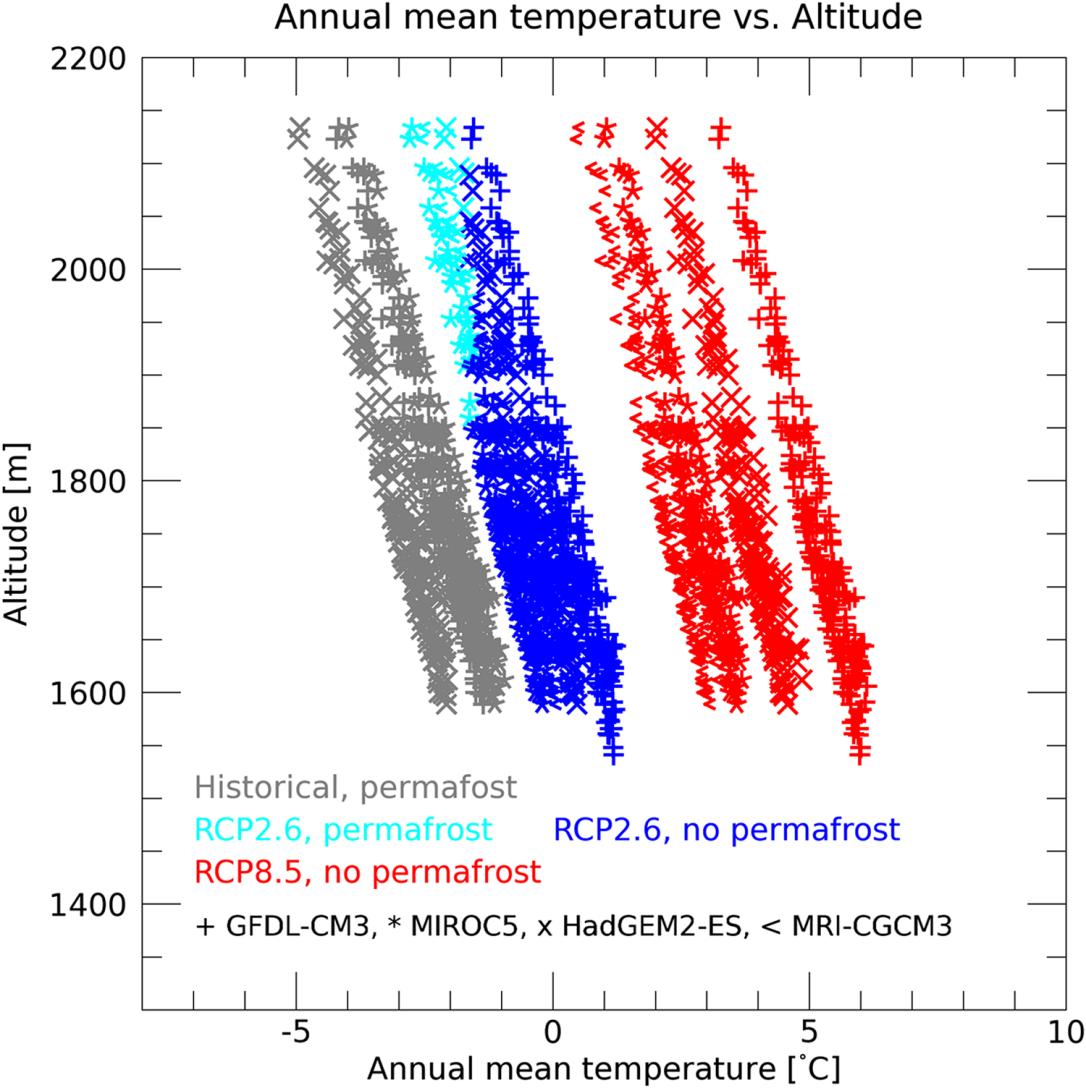
Scatter plot of altitude-annual mean surface air temperature in the Daisetsu Mountains. Each point represents the output of an individual grid cell. Only grid cells where the climatic conditions suitable for the development of permafrost (environmentally conditioned permafrost, ECP region) in the historical simulations are plotted (2001–2010: gray). These points are plotted separately in the ECP region (cyan) and the non-ECP region (blue) in the year 2100 under the RCP2.6 scenario. Since there is no ECP region in the year 2100 under the RCP8.5 scenario, all of the points are plotted in red. The symbols represent outputs from the four global climate models of the bias-corrected climate scenarios.

Figure 4 shows future changes in surface air temperatures at the grid points for the ECP regions under the current climate conditions (i.e., at the grid points where the value is $>0$ in Fig. 2). Based on historical projections, the lower altitude limit for the ECP regions is estimated to be approximately 1600 m, but it is approximately 1900 m at the end of the twenty-first century under the RCP2.6 scenario based on future projections (Fig. 4, light blue). In both the historical and RCP2.6 projections, the upper limit of the annual mean surface air temperature in the ECP regions is approximately − 2 °C. The altitude at which this temperature is achieved is projected to increase by approximately 300 m by the end of the twenty-first century under the RCP2.6 scenario, which implies a severe reduction in the ECP regions. Under the RCP8.5 scenario, the annual mean surface air temperature is projected to exceed 0 °C by the end of the twenty-first century, even at the highest altitudes (> 2100 m), resulting in the complete disappearance the ECP regions.

**Figure 5:**
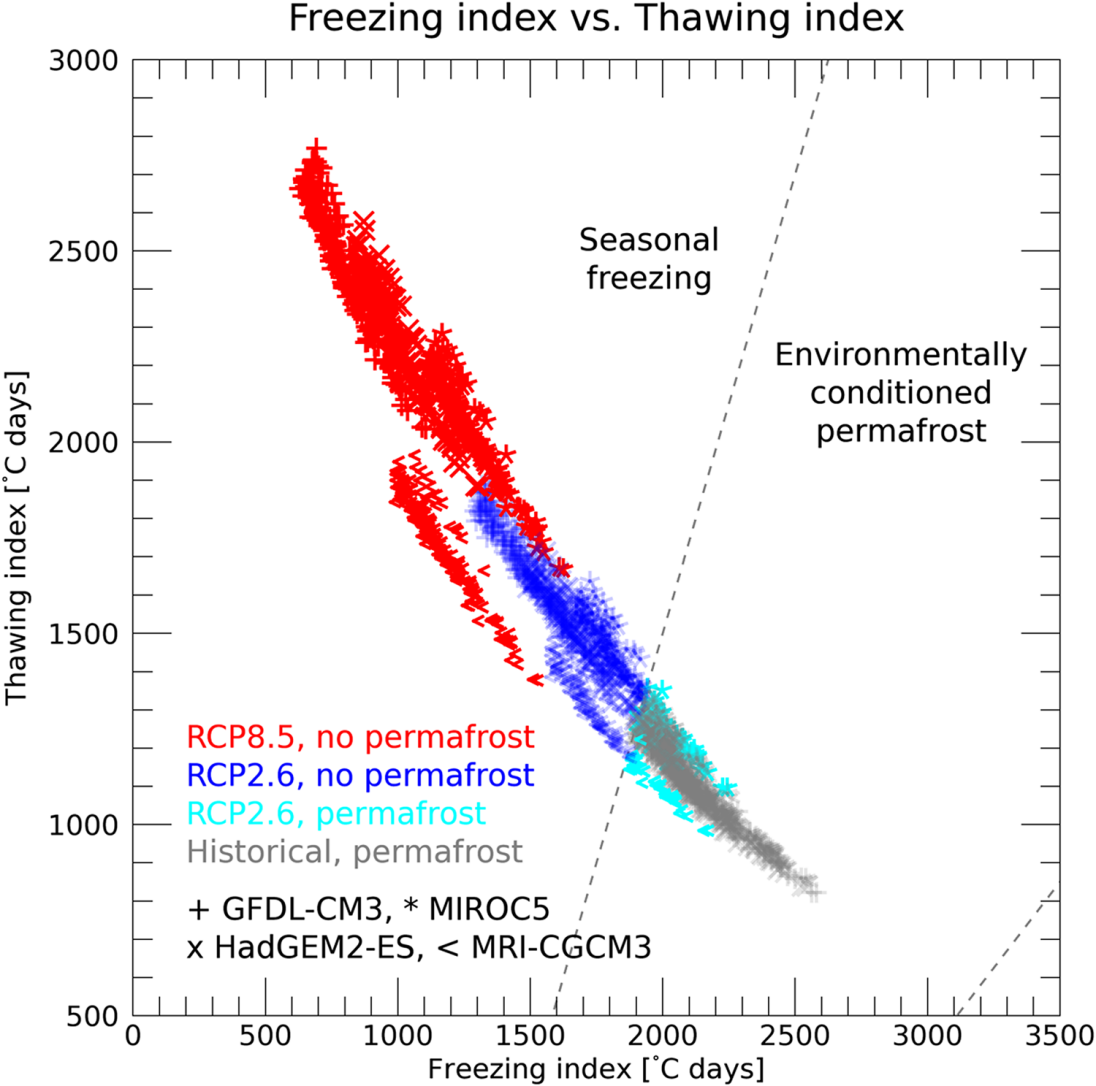
Scatter plot of freezing and thawing indices in the Daisetsu Mountains. The method used to produce the plot is the same as that in Fig. 4. Bias-corrected climate scenarios based on the four global climate models are used to calculate the freezing and thawing index averaged over the last 30 years. Areas of “Environmentally conditioned permafrost” and “Seasonally freezing” follow the definitions given in "Statistical method for inferring permafrost distribution" section.

Figure 5 shows the freezing and thawing indices for the ECP regions under present climate conditions. The grey data points represent the ECP regions under the current climate conditions in the Daisetsu Mountains. At the end of the twenty-first century, under both the RCP2.6 and RCP8.5 scenarios, the freezing index decreases and the thawing index increases, shifting the freezing–thawing index scatterplot to the upper left. A small ECP region is observed under RCP2.6 conditions, but all of the data points have shifted to the seasonally freezing region under RCP8.5 conditions.

**Figure 6:**
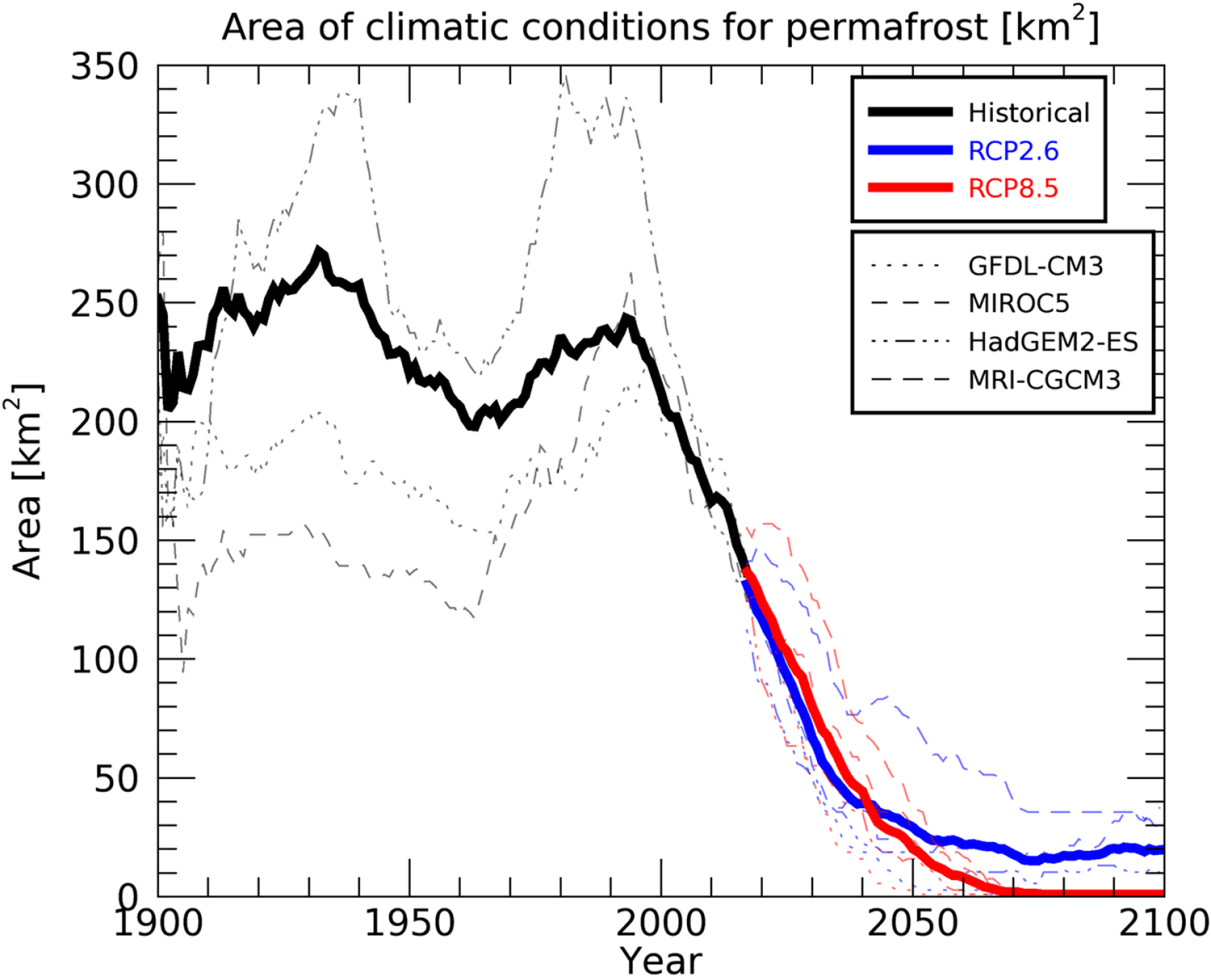
Time series of the area where climatic conditions are suitable for the maintenance of permafrost (environmentally conditioned permafrost, ECP) in the Daisetsu Mountains. Results of historical (black) and future projections under the RCP2.6 (blue) and RCP8.5 (red) scenarios are shown. Thin broken lines show the results obtained from the four bias-corrected climate scenarios based on different global climate models, and the thick line is the average of the results using the four bias-corrected scenarios. The ECP region was calculated using the average value of the freezing and thawing indices over a 30-year period.

Figure 6 shows the time series of the ECP region in the Daisetsu Mountains. As shown in Fig. 2, the ECP region is widely distributed in the historical experiments, and the average value for the bias-corrected climate scenarios from the four GCMs is about 150 km^2^ as of 2010 (corresponding to the blue area in Fig. 3). During the historical simulation period (1900–2018) shown in Fig. 6, long-term variations are observed, indicating that the surface air temperature in Japan varies on a scale of several decades. Another major feature of the historical simulation results is that the ECP region varies markedly depending on the four types of bias-corrected climate scenarios. The large spread in the ECP region in the historical simulations is because surface air temperatures differ depending on the bias-corrected climate scenarios (Figure S3). Since the ECP region is calculated using the average value for the past 30 years, Figure S2 is also calculated using the average value for the past 30 years. As shown in Figure S2, the surface air temperature varies greatly during the twentieth century, depending on GCMs.

On the other hand, the spread in the ECP regions decreased at the beginning of the twenty-first century (Fig. 6) because the model bias was corrected with reference to the observational data from 1980 to 2018. With the bias correction, the monthly mean of the bias-corrected climate scenarios averaged over 1980–2018 matches the observed data during this period. In the results for changes in the surface air temperature (Figure S3), the range in the surface temperatures is relatively small near the reference period (1980–2018).

**Figure 7:**
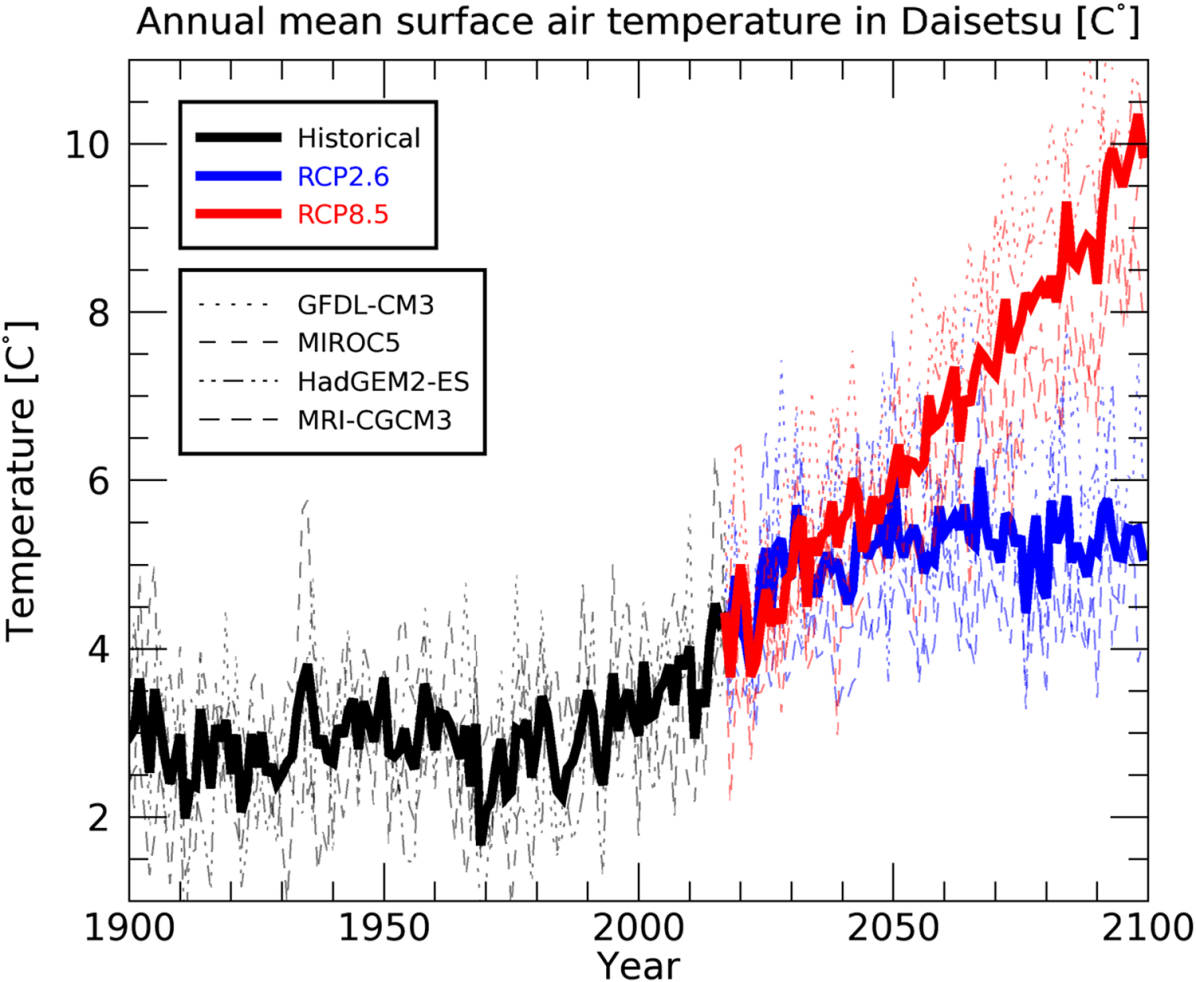
Same as Fig. 6 but the annual mean surface air temperature averaged over Daisetsu Mountains region as shown in Fig. 1c.

According to Fig. 6, the ECP region starts to decrease significantly in size from around 2000, and under both RCP2.6 and RCP8.5 scenarios, it decreases to approximately 30 km^2^ in 2050. The steep decline in the ECP region at the end of the twentieth century occurs because the surface air temperature increases significantly from around the year 2000 onward (Figure S3). Figure 7 shows the annual mean surface air temperature in the Daisetsu Mountains region (area shown in Fig. 1c). Compared to the global average (Fig. 3), the average over the Daisetsu Mountains region shows a larger increase in annual mean surface air temperature. This large increase likely occurred because the Daisetsu Mountains region is located at a relatively high latitude (43–44°N) and is affected by polar amplification through processes such as snow and ice albedo feedback^64^. The annual mean surface air temperature in the Daisetsu Mountains region was about 3℃ during the twentieth century, but the average surface air temperature of the four bias-corrected GCMs is projected to rise to about 10 °C by the end of the twenty-first century under the RCP8.5 scenario. Such increases in the surface air temperature are projected to reduce the area of permafrost regions in the Daisetsu Mountains (Fig. 6).

As shown in Fig. 6, the decrease in the ECP region under the RCP2.6 scenario slows down due to stabilization of the global mean average temperature (Fig. 3); however, only a small amount of the ECP region remains in 2100 (approximately 20 km^2^). On the other hand, under the RCP8.5 scenario, all four types of bias-corrected climate scenarios show that the ECP region is projected to disappear completely by around 2070. These results are consistent with future projections of mountain permafrost in Europe^8^ and on the Tibetan Plateau^11^.

**Figure 8:**
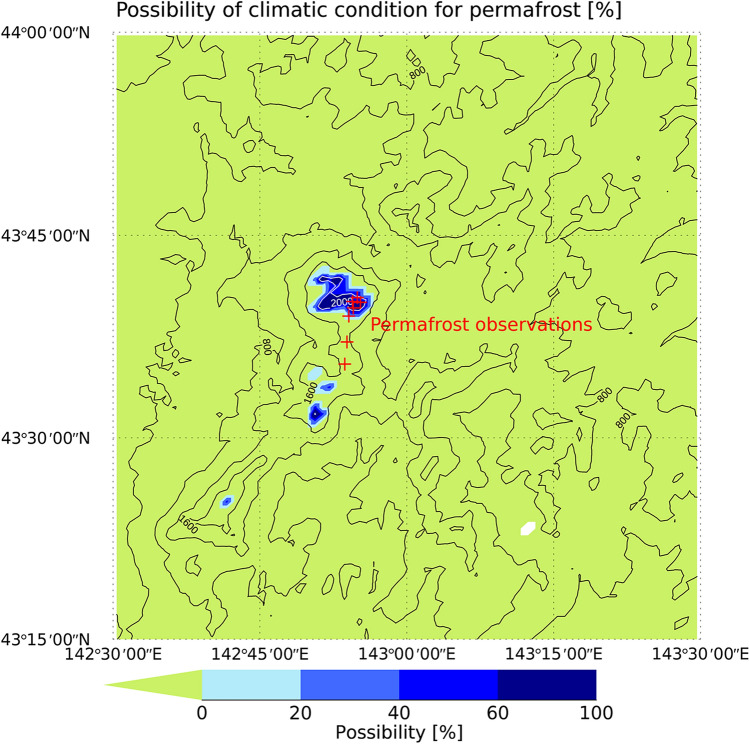
Same as Fig. 3, but the results for the RCP2.6 scenario for 2100 are shown. Contour lines show altitude distribution. Contour lines above and below 2000 m are shown in white and black, respectively. IDL (https://www.l3harrisgeospatial.com/Software-Technology/IDL) is used to plot this figure.

Finally, Fig. 8 shows the distribution of the ECP region at around 2100 under the RCP2.6 scenario (average of the four climate scenarios for 2091–2100). As can be seen, the region is limited to altitudes above 2000 m in 2100. Compared to the ECP region under the present climate conditions (Fig. 2), this represents a significant decrease, and the ECP disappears completely from the two observation points near 1700 m.

## Discussion and conclusions

In the present study, the ECP regions corresponding to the areas where permafrost can exist were estimated using historical and future projections, based on the freezing and thawing indices^52^ and 1-km resolution bias-corrected climate scenarios for Japan^54^. In the ECP regions, the presence of permafrost is dependent upon environmental factors such as ecosystem characteristics, topography and/or geology. The historical and future projections of bias-corrected and downscaled climate model outputs used in this study are based on numerical experiments performed using four different GCMs. Therefore, it is possible to consider uncertainties in future climate projections^65^, and it is also expected that issues related to errors that are peculiar to the GCMs can be alleviated to some extent. On the other hand, future projections of permafrost area could be affected by internal climate variability, such as Atlantic Multidecadal Variability, and thus analysis using a large ensemble simulation output is an important research topic for the future. In Japan, mountain permafrost has been observed in the Daisetsu Mountains, on Mt. Fuji and on Mt. Tateyama. We focused on the Daisetsu Mountains because observational studies have already been conducted on the permafrost in this area.

According to our estimates, the ECP region in the Daisetsu Mountains in 2010 is approximately 150 km^2^ in size (average of four bias-corrected climate scenarios); however, marked variations were observed depending on the GCM used. We confirmed that permafrost has been observed at points that were estimated to be ECP regions (Fig. 2), suggesting that our estimation results corroborate observations made in the field.

According to our analysis, ECP areas are projected to decline rapidly after 2000 (Fig. 6). From the historical simulation results, the lower altitude limit of the ECP region was approximately 1600 m, but it is projected to rise to approximately 1900 m by 2100 under the RCP2.6 scenario (Fig. 4). The ECP region is also projected to be reduced to approximately 20 km^2^ by 2100 under the RCP2.6 scenario (Fig. 6). Under the RCP8.5 scenario, the ECP region is projected to disappear entirely from the Daisetsu Mountains by around 2070 (Fig. 6).

It should be noted that the analysis presented in this study does not consider detailed factors such as topography (slope direction and slope angle), soil properties (porosity and permeability), and micro-meteorological conditions (local wind direction and snow cover) that are known to play important roles in determining the distribution of permafrost^28^. The statistical classification method developed by Saito et al.^52^ was based on ground freezing conditions observed at 571 sites ranging from high- (i.e., Alaska and Siberia) to mid-latitude (i.e., China and Mongolia) sites; the wide range of sites selected in their study ensured that both cold and warm permafrost sites with a variety of local conditions were incorporated into their analysis (Fig. 2 of Saito et al.^52^). However, since the method assumes a quasi-equilibrium in the relationship between the surface air temperature and ground freezing, it may behave differently under the abruptly changing climate conditions expected in the future. As a future study, it will be important to refine their method by including the newly observed changes, as well as to examine the permafrost distribution at a high spatial resolutions by developing an improved land surface model^42^ that adequately describes the physical state of the ground.

Using a 30-year average as the calculation period for the freezing and thawing indices, hysteresis in surface air temperature changes is considered in our estimates of the ECP region (see "Statistical method for inferring permafrost distribution" section). However, the response of the permafrost to the changes in climatic conditions may occur over a much longer time scale^52^, and changes in permafrost distributions may be slower than those shown in Fig. 6. Nonetheless, the extent of permafrost in the Daisetsu Mountains is projected to decrease significantly due to future rises in temperature.

### Importance of adaptation to climate change in the Daisetsu Mountains

The finding that the climatic conditions of the Daisetsu Mountains are projected to shift towards conditions where permafrost will disappear regardless of the climate scenario used (i.e., RCP2.6 and RCP8.5; Fig. 6), indicates the importance of impact assessments and developing adaptation measures to climate change. Since frozen soil has a low permeability, infiltration of water is prevented at the upper surface of the permafrost layer. Also, soil moisture is maintained in the surface layer, called the active layer^66^. This active layer freezes and thaws on a seasonal basis, and alpine plant communities have adapted to these conditions^67^. Consequently, thawing of permafrost can have a significant impact on the surface vegetation and alpine ecosystems^68^. Previous studies have shown that the thawing of permafrost can have a variety of adverse effects, including lowering groundwater levels and replacement of alpine meadows by steppes^20^, replacement of hygrophilic plant communities by xeromorphic communities or shrubs^21^, extensive desertification such as that in the eastern and western parts of the Tibetan Plateau^69^, and a decrease in species diversity due to warming^70^.

Several research teams are currently monitoring the phenology and distribution of alpine vegetation in the Daisetsu Mountains^71^. While observations of alpine vegetation in the Daisetsu Mountains have not yet clarified the effect of changes in permafrost, it is important to study the effects of changes in permafrost on this alpine ecosystem. For example, future studies should compare locations where the distribution of permafrost is likely to change and locations where it is not. In addition, surveys of physical parameters, such as soil moisture and temperature, and how these affect permafrost dynamics should be undertaken in conjunction with monitoring alpine vegetation. Alpine plants, flower meadows, and snowy gorges (snow-covered valleys) are valuable tourism resources, and the changes and loss of these environments will have a negative impact on tourism demand (Kubo et al. in preparation). It is therefore necessary to carefully manage these resources in response to any changes in tourism demand that may be caused by changes in climate and permafrost.

Changes in the alpine ecosystem associated with changes in the frozen ground regime may also affect the behavior of wildlife, such as the foraging habits and range of activity of animals. While wildlife is an invaluable tourism resource in the alpine zone, the risk of encounters between mountaineers and wildlife is also an important consideration^33,34^. It has also been reported that human activities can adversely affect the range of wildlife activities^35^, and changes may need to be implemented in order to manage wildlife in mountainous areas in response to the impacts of climate change.

In addition, thawing of the frozen ground reduces the stability of the ground in mountain regions, potentially increasing the frequency and magnitude of rock falls and landslides^72^, which may affect the safety of trekkers that visit the Daisetsu Mountains annually. In order to deal with this problem, it is very important to monitor the environmental changes in mountainous areas. In recent years, a number of case studies focusing on ground-surface displacement in permafrost regions using satellite microwave data have been reported^73,74^. Since the methods employed in these studies can capture ground movements at the scale of several centimeters in a year, it may be possible to identify locations where slope displacement and landslides are most likely to occur (Iwahana et al. in preparation). If such analyses reveal that there is a high risk of slope displacement on an existing mountain trail, then the mountain trekking routes will need to be changed. This could greatly aid decisions on whether to maintain the trails in an area and how much to invest on route maintenance and improvement. Collecting donations from hikers is also an effective way to maintain the trails^35^. In addition to accurately monitoring changes in mountain environments, providing local governments with appropriate measures to prepare for major future environmental changes, as shown in this study, is an important issue for the future.

## Methods

### Bias-corrected and downscaled climate model output

In this study, we used bias-corrected and downscaled climate model outputs developed by Ishizaki et al.^54^, who generated two bias-corrected climate scenarios using different methods. We used the climate scenarios based on the cumulative distribution function-based downscaling method (CDFDM, developed in previous studies^75–77^). Using the CDFDM, the cumulative distribution function for simulated daily mean data is corrected so that it matches the 1 km-resolution meteorological data for Japan^58^. Ishizaki et al.^54^ demonstrated that the CDFDM is superior to other methods, such as Gaussian-type scaling approaches^78^. Further, Ishizaki et al.^54^ corrected biases in historical simulations and future projections based on the RCP2.6 and RCP8.5 scenarios for four GCMs (GFDL-CM3^79^, MIROC5^80^, HadGEM2-ES^81^, MRI-CGCM3^82^, Nor-ESM^83^) for the Japan region at a resolution of 1 km. Briefly, the reasons why Ishizaki et al.^54^ selected four global climate models from the CMIP5 global climate models was so that the models could cover the uncertainty ranges of future surface air temperature and precipitation projections, and because of their reproducibility in the twentieth century climate. The reproducibility of the twentieth century climate projections was evaluated using climate metrics^84^. Of the four models, two (MIROC5 and MRI-CGCM3) were selected because they were developed in Japan and are often used in studies on climate change around Japan^54^.

Ishizaki et al.^54^ showed that the bias-corrected historical climate scenarios accurately reproduced monthly averaged values; extreme values, such as summer days; and indicators defined by daily values, such as precipitation intensity. In this study, we utilized ver. 202005 in which the time window for the cumulative distribution function (one month) and the reference period (1980–2018) were modified so that the monthly values corresponded to those of the observations.

### Statistical method for inferring permafrost distribution

We employed the method of Saito et al.^52^ to infer the permafrost distribution using the bias-corrected climate scenarios. Saito et al.^52^ classified permafrost using a freezing index, i.e., the number of days per year when the surface air temperature is below 0 °C multiplied by the surface air temperature, *I*_f_, and the thawing index, i.e., the number of days per year when the surface temperature is above 0 °C multiplied by the surface air temperature, *I*_t_, as follows:

- Climate-driven permafrost: CDP
  1 $${I}_{t}<{0.9 I}_{f}-2300$$
- Environmentally conditioned permafrost: ECP
  2 $$ 0.9 I_{f} - 2300 < I_{t} < 2.4 I_{f} - 3300 $$
- Seasonal freezing: SF
  3 $$ 2.4 I_{f} - 3300 < I_{t} \quad {\text{and }}\quad 30 < I_{f} $$
- Intermittent freezing: IF
  4 $$0 <{I}_{f} \le 30$$

For consistency with Saito et al.^52^, this study uses the monthly mean surface air temperature to calculate the freezing and thawing indices. Previous studies have shown that the relative error is less than 5% when using daily or monthly means^59^. Saito et al.^52^ performed a permafrost classification at a resolution of 2 km by considering the temperature decrease with altitude using spatially detailed elevation data (ETOPO1^60^) based on the results of the CMIP5 GCMs. In the present study, a temperature decrease with altitude was considered in the 1-km mesh observational data^58^ used for bias-corrected climate scenarios.

In this study, the freezing and thawing indices for the past 30 years were averaged to classify the permafrost in each grid, as it was done in Saito et al.^52^. Averaging over 30 years smoothens the internal variability in surface air temperature and corresponds to the delayed response of the permafrost to climate change.

## Supplementary Information

Supplementary Information.

## Data Availability

Data sharing is not applicable to this article. Please contact the authors for data requests.
