# Supplementary material for: Projections of surface air temperature required to sustain permafrost and importance of adaptation to climate change in the Daisetsu Mountains, Japan
Source: Sci Rep. 2021 Jul 30;11:15518. doi: 10.1038/s41598-021-94222-4 (PMC8324894; doi:10.1038/s41598-021-94222-4)
Supplement: Supplementary file 1 — Supplementary Information. [file 41598_2021_94222_MOESM1_ESM.docx]

**Projections of surface air temperature required to sustain permafrost and importance of adaptation to climate change in the Daisetsu Mountains, Japan**

Tokuta Yokohata^a*^, Go Iwahana^b,a^, Toshio Sone^c^, Kazuyuki Saito^d^, Noriko N. Ishizaki^a^, Takahiro Kubo^a^, Hiroyuki Oguma^a^, Masao Uchida^a^

^a^ *National Institute for Environmental Studies, Tsukuba, Japan*

^b^ *University of Alaska Fairbanks, Alaska, USA*

^c^ *Hokkaido University, Sapporo, Japan*

^d^ *Japan Agency for Marine-Earth Science and Technology, Yokohama, Japan*

**Table S1** Locations of permafrost occurrence based on ground temperature measurement or estimated from the basal temperature of snow (BTS).

| **No** | **Name** | **Altitudes** | **Reference** |
| --- | --- | --- | --- |
| 1 | Mt. Hakuundake | 2170m | Iwahana et al.^57^ |
| 2 | Mt. Koizumidake | 2150m | Iwahana et al. ^57^ |
| 3 | Hokkaidaira | 2075m | Fukuda and Sone^84^ |
| 4 | Mt. Goshikidake | 2035m | Iwahana et al.^59^ |
| 5 | Mt. Chubetsudake | 1850m | Ishikawa and Hirakawa^28^ (BTS) |
| 6 | Mt. Takanegahara | 1755m | Sone and Watanabe^59^ |
| 7 | Mt. Hiragatake | 1720m | Sone and Takahashi^85^, Sone^87^ |

**
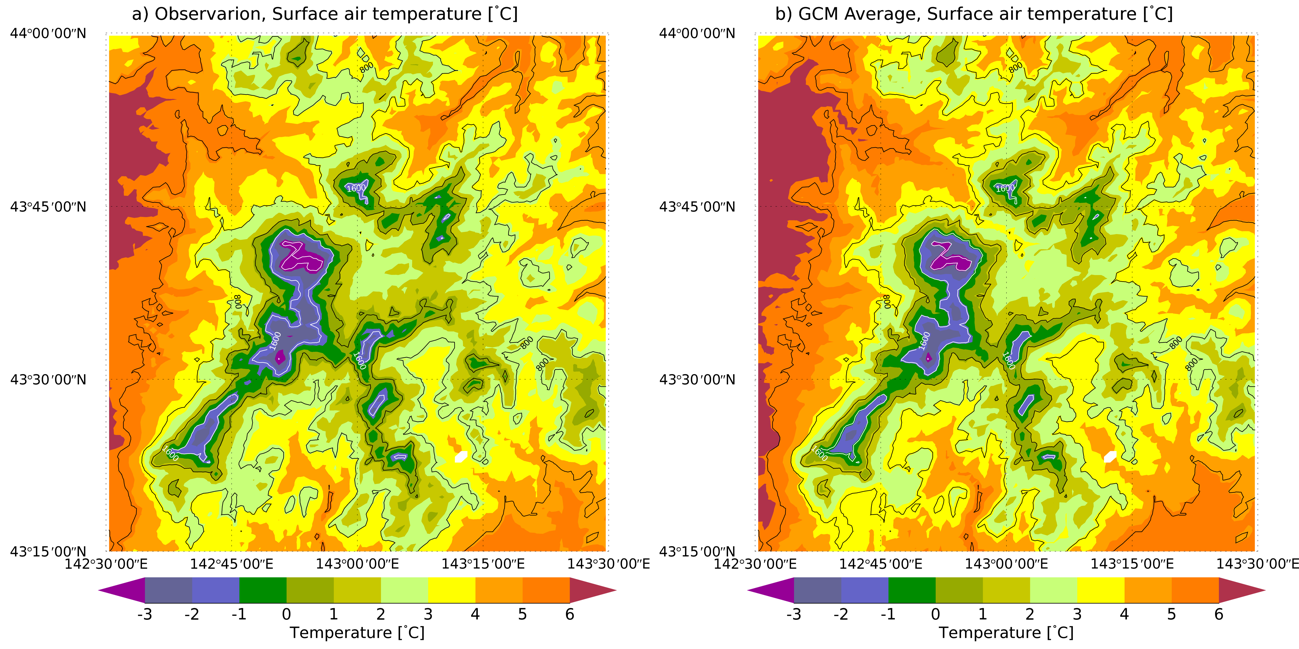
**

**Figure S1.** Average surface air temperature for 2001–2010 in the Daisetsu Mountains, a) meteorological dataset by Ohno et al.^58)^, and b) average of four bias-corrected climate scenarios (same as Figure 1c).

**
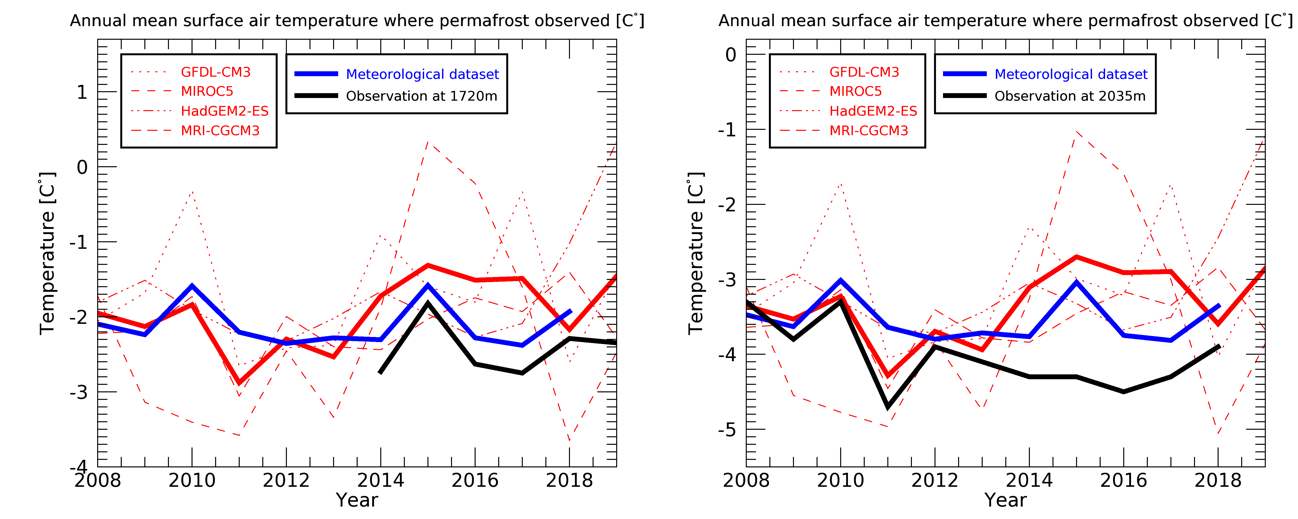
**

**Figure S2.** Time series of annual mean surface air temperature at locations where permafrost has been observed in the Daisetsu Mountains. Thin dashed/dotted lines (red) show the results of four bias-corrected global climate models (GCMs), and thick solid lines (red) show the averages of the four bias-corrected GCMs. The blue line shows the meteorological data^58^, and black line shows the field observational data at the observation site of a) Mt. Goshikidake (2035 m)^59^, and b) Mt. Hiragatake (1720 m)^86^. For the calculation of bias-corrected GCMs, we chose the nearest grid points of the observational site, and made an altitude correction (0.65℃/100 m) by using the difference in altitudes between the GCM’s grid point and observational site.

**
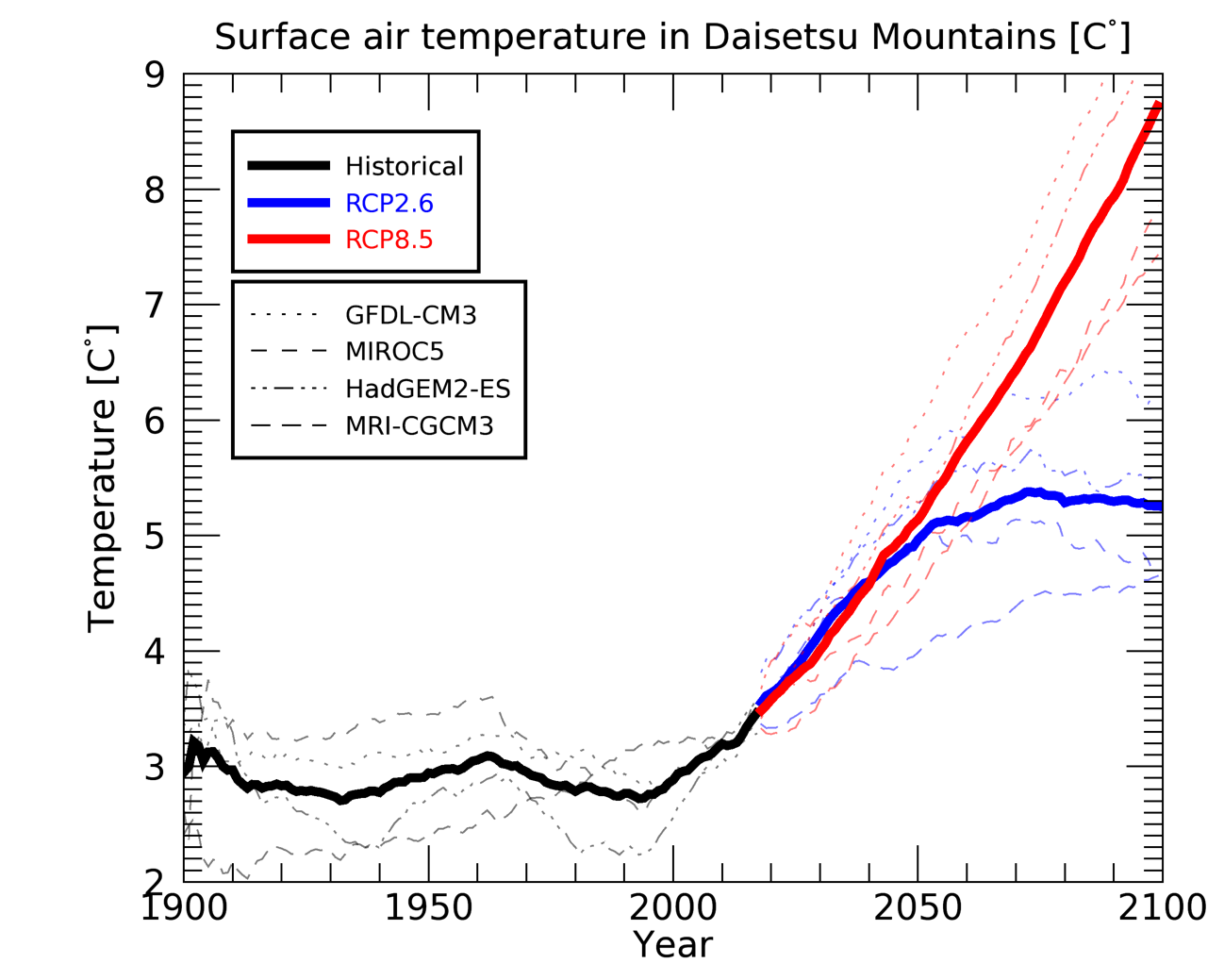
**

**Figure S3.** Time series of surface air temperatures in the Daisetsu Mountains for the historical (black), the RCP2.6 (blue), and RCP8.5 (red) scenarios. Thin dotted/dashed lines show the results of the four global climate models (GCMs), and the thick line is the average of the four GCMs. The surface air temperature is an average for the past 30 years.
